# Recent advances in understanding and managing malabsorption: focus on microvillus inclusion disease

**DOI:** 10.12688/f1000research.20762.1

**Published:** 2019-12-05

**Authors:** Dulari Jayawardena, Waddah A. Alrefai, Pradeep K. Dudeja, Ravinder K. Gill

**Affiliations:** 1 Division of Gastroenterology & Hepatology, University of Illinois at Chicago, Chicago, IL, USA; 2 Jesse Brown VA Medical Center, Chicago, IL, USA

**Keywords:** MVID, malabsorption, epithelial transport, diarrhea, trafficking

## Abstract

Microvillus inclusion disease (MVID) is a rare congenital severe malabsorptive and secretory diarrheal disease characterized by blunted or absent microvilli with accumulation of secretory granules and inclusion bodies in enterocytes. The typical clinical presentation of the disease is severe chronic diarrhea that rapidly leads to dehydration and metabolic acidosis. Despite significant advances in our understanding of the causative factors, to date, no curative therapy for MVID and associated diarrhea exists. Prognosis mainly relies on life-long total parenteral nutrition (TPN) and eventual small bowel and/or liver transplantation. Both TPN and intestinal transplantation are challenging and present with many side effects. A breakthrough in the understanding of MVID emanated from seminal findings revealing mutations in *MYO5B* as a cause for MVID. During the last decade, many studies have thus utilized cell lines and animal models with knockdown of *MYO5B* to closely recapitulate the human disease and investigate potential therapeutic options in disease management. We will review the most recent advances made in the research pertaining to MVID. We will also highlight the tools and models developed that can be utilized for basic and applied research to increase our understanding of MVID and develop novel and effective targeted therapies.

## Diagnosis of microvillus inclusion disease

In 1978, Davidson *et al.* presented a case report of five infants with persistent severe diarrhea from birth and marked abnormalities of absorption associated with failure to thrive, leading to death in four infants ^1^. The common histological abnormalities in duodenal mucosa from those infants were villus atrophy, crypt hypoplasia (without an increase in mitoses or inflammatory cell infiltrate in the lamina propria) and absence of a brush border in villus enterocytes, and an increase in lysosome-like inclusions ^2, 3^. Originally referred to as Davidson's disease, congenital microvillus atrophy, and intestinal microvillus dystrophy, the disease was named microvillus inclusion disease (MVID) in 1989 by Cutz *et al*. ^4^.

As with all rare genetic diseases, the diagnosis of MVID was quite challenging until recently and required histological evaluation for confirmation. It is important to note that MVID has a very low incidence, making it extremely difficult to investigate its pathophysiology ^5^. The morphological anomalies observed in the enterocytes of patients with MVID are widely utilized in disease diagnosis ^2^. Until recently, the gold standard in diagnosing MVID was combined light and electron microscopy of small bowel biopsy samples of patients. The abnormalities are mainly observed in the small intestine and less frequently in the colon ^6^. However, some studies have shown that the colon and rectum biopsies may also contain characteristic features which would be useful in diagnosing MVID ^2, 6, 7^.

**Figure 1.**
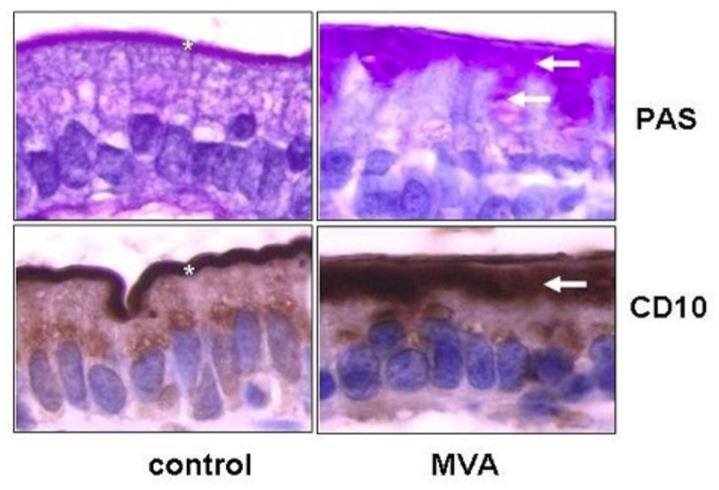
Characteristic histological features of microvillus inclusion disease reprinted with permission from Ruemmele *et al*. ^2^. MVA, microvillous atrophy; PAS, periodic acid Schiff

The key hallmarks which aid in the differential diagnosis include blunted or absent microvilli, accumulation of secretory granules, and microvillus inclusions (MIs) in the epithelial cells ^2, 8^. As depicted in Figure 1, these granules, in most cases, are positive for periodic acid Schiff (PAS) stain and CD10 with an intracellular PAS or CD10 positive line in enterocytes that is commonly detected. Another apical marker which may aid in the identification of the trademark MIs is villin, an apical surface marker of enterocytes ^9^. An important factor which should be accounted for during histological evaluation of biopsies is sampling variability and patient-to-patient variability. The diagnosis is confirmed further by genetic testing, which can specifically identify the genetic anomaly of each patient. In this instance, currently, there is a registry which tracks each genetic variation observed in MVID patients to facilitate ease of access to patient-related data for clinicians involved in the management of this rare genetic disorder ^10^.

## Differential diagnosis

There are several features that differentiate MVID from other diarrheal conditions with similar clinical presentation including the onset at birth, absence of inflammation, presence of vacuoles containing granules with the characteristic PAS and CD10 positive stain observed under light microscopy, and presence of MIs ( Table 1). Other congenital disorders such as chloride and sodium diarrhea can be easily excluded from biochemical assays or genetic testing ^6, 11^. Tufting enteropathy is a disorder with similar onset and blunted villi; however, the presence of surface apical tufts as opposed to apical inclusion bodies in enterocytes distinguishes tufting enteropathy from MVID. Enteroendocrine cell dysgenesis can be differentiated from MVID by the lack of enteroendocrine cells and the presence of normal microvilli. Finally, abetalipoproteinemia is distinguished from MVID by the presence of fat vacuoles and a foamy cytoplasm ^20^.

**Table 1.** Characteristic features of congenital diarrheal disorders.

Congenital disease	Major gene/s mutated	Distinctive feature(s)
Microvillus inclusion disease	*MYO5B* ^12^, *STX3* ^13^, *STXBP2* ^14^	Blunted microvilli, microvillus inclusions
Chloride diarrhea	*SLC26A3* or Down Regulated in Adenoma ( *DRA*) ^15^	High-chloride diarrhea (fecal Cl ^–^ >90 mM/L) and normal microvilli
Sodium diarrhea	*SPLINT2* (serine peptidase inhibitor 2), *GUCY2C* (guanylate cyclase C), and *SLC9A3* (sodium hydrogen exchanger 3 (NHE3) ^16^	High-sodium diarrhea (fecal Na ^+^ >145 mM/L) and normal microvilli
Tufting enteropathy	*EPCAM* (epithelial cell adhesion molecule) ^17^	Presence of surface apical tufts with blunted villi
Enteroendocrine cell dysgenesis	*NEUROG3* (neurogenin-3) ^18^	Lack of enteroendocrine cells with normal villi
Abetalipoproteinemia	*MTTP* (microsomal triglyceride transfer protein) ^19^	Fat vacuoles with foamy cytoplasm and normal villi

## Clinical manifestations of microvillus inclusion disease

Earlier studies in patients with MVID showed high stool volume (150 to 300 mL/kg/day) with remarkably elevated sodium content (approximately 100 mmol/L) ^2, 21^. The diarrhea present in MVID is considered to be non-osmotic in nature (i.e. fecal ion gap <100 mOsm) and is persistent even when the patient is unfed ^22^. This type of diarrhea is categorized as electrolyte transport-related diarrhea caused by mechanisms involving net secretion of anions (chloride, bicarbonate, or potassium) and/or net inhibition of sodium or chloride absorption ^11, 23– 25^. Steatorrhea and impaired glucose absorption have also been reported in MVID patients ^26, 27^. Various studies have shown mislocalization of apical membrane-targeted proteins such as sucrase isomaltase, alkaline phosphatase, and sodium hydrogen exchanger 3 (NHE3) in MVID, which might partly explain the pathophysiology of malabsorption and diarrhea ^28^. Due to the high-volume and persistent diarrhea observed in these patients, the main life-saving treatment option remains life-long total parenteral nutrition (TPN). The use of life-long TPN poses many complications including sepsis and worsening cholestatic liver disease that may require intestinal transplantation. However, outcomes of intestinal transplantation remain poor ^2, 27^. Owing to the immature nature of the enterocytes present in infants with MVID, absorption of essential nutrients is hampered and, therefore, recent studies are directed at developing therapeutic agents which are capable of increasing the maturity of the enterocytes to ultimately recuperate the loss of absorptive capacity of the small intestine ^29^. Although little progress has been made in developing treatment options, the growing research has certainly highlighted relevant mechanisms linking perturbation in cellular trafficking and signaling pathways to functional physiological defects leading to malabsorption and chronic diarrhea ^30^.

## Pathophysiology of microvillus inclusion disease

### Studies in patients and human cell lines

The identification of gene mutations linked to trafficking pathways in MVID has paved the way for further research into better understanding of this intricate and challenging enteropathy. The major mutation observed in MVID patients is in the *MYO5B* gene, the key molecular motor gene regulating trafficking of important proteins into the brush border of the intestinal epithelial cells ^31^. An online registry for MVID patients and their mutations has been generated which currently has 188 MVID patients ^10^. Although the majority of MVID patients exhibit mutations in *MYO5B*, mutations in other genes have also been identified that present with less severe enteropathy. For example, mutations in soluble N-ethylmaleimide-sensitive factor attachment protein receptor (SNARE) protein syntaxin-3 ( *STX3*) cause a variant form of MVID with lateral microvilli and occasional microvillus occlusions ^13^. In addition, patients with mutations in *STXBP2,* encoding the syntaxin-binding protein-2 (MUNC18-2) protein, also have intestine-related hallmarks of MVID besides their primary diagnosis of familial hemophagocytic lymphohistiocytosis type 5 (FHL5), a hyper-inflammatory immune disorder ^14^. Recent studies by Dhekne *et al*. ^32^ provided further evidence that *MYO5B*, *STX3*, and *STXBP2* genes are functionally linked in MVID patients. In this regard, analysis of subcellular distribution of STX3 and MUNC18-2 in enterocytes of intestinal biopsies from patients with *MYO5B* or *STXBP2* mutations showed that MUNC18-2 and STX3 accumulated in intracellular puncta in the enterocytes of MVID patients as compared to apical localization in brush border plasma membrane in control enterocytes. In addition to the native biopsy samples, *in vitro* Caco2 model epithelium has been used extensively to recapitulate the loss of MYO5B on epithelial polarity and intracellular trafficking. Interestingly, *MYO5B* knockdown mimicked the loss of apical microvilli and lack of polarity and was associated with internalization of several apical membrane transporters such as Na ^+^/H ^+^ exchanger NHE3 ^31, 33, 34^ and Down Regulated in Adenoma (DRA) ^34^. While both NHE3 and DRA localization were significantly reduced on the apical membrane of human MVID enterocytes and *MYO5B* knockdown ( *MYO5B*-KD) C2BBe cells, the localization of cystic fibrosis transmembrane conductance regulator (CFTR) was mostly preserved ^28^. Functional studies confirmed that Forskolin-stimulated CFTR ion transport was intact in *MYO5B*-KD T84 cells ^28^.

Another recent study using stable *MYO5B*-KD in CaCo2-BBE cells established the critical role of MYO5B interactions with specific RAB small GTPases (RAB8A and RAB11) in MVID ^35^. *MYO5B*-KD cells showed loss of microvilli; however, no MIs were observed. The expression of WT MYO5B in *MYO5B*-KD cells restored microvilli, while the expression of MYO5B–P660L, an MVID-associated mutation found within the Navajo population (that cannot bind to RAB11A), induced the formation of MIs but did not rescue the *MYO5B*-KD phenotype. On the contrary, the expression of a RAB8A binding-deficient MYO5B mutant partly restored the microvilli loss, but no inclusions were formed. These studies demonstrated that the disruption of the MYO5B–RAB11A interaction results in the formation of MIs, whereas MYO5B–RAB8A binding is important for microvilli formation ^35^. Recent studies by Vogel *et al*. identified Rab11- and/or Rab8-positive recycling endomembrane compartments that were enriched with apical membrane proteins, including STX3 and NHE3, in MVID patients’ enterocytes ^36^.

With respect to mechanisms underlying the origin of inclusions and microvillus loss, a recent review by Schneeberger *et al*. ^29^ highlighted three potential models or a combination of these models to explain the pathological hallmarks of MVID. In the first, described as a trafficking model, defects in vesicle trafficking caused by *MYO5B* or *STX3* mutations result in the subapical accumulation of vesicles and in the lack of appropriately polarized apical proteins. In the second model (recycling model), perturbations in the recycling and delivery of apical recycling endosomes (AREs) result in the subapical accumulation of apical proteins and in the formation of microvilli-containing macropinosomes. As discussed above, MYO5B is required for the localization of RAB11A-positive AREs, which contain various signaling molecules, such as pyruvate dehydrogenase kinase (PDK1), protein kinase C (PKCi), and serine threonine protein kinase (MST4) colocalized with ezrin ^28, 32, 37^. The third local induction model proposes that in MVID, RAB11A-positive AREs accumulate and function as a subapical signaling platform to induce ectopic intracellular microvillus formation ^37^. The presence of MIs in MVID is the pathognomonic finding based on microscopy of intestinal tissues in diagnosing patients. However, the formation of these inclusions in enterocytes is not yet defined as a cause or consequence of the disease, although the latter is more accepted in the current clinical setting. Plausibly, MIs may represent a secondary effect of overall disrupted epithelial polarity in MVID ^38^.

### Animal models to study microvillus inclusion disease

In the first report of animal models of MVID initiated about 4 years ago, in 2015, Schneeberger *et al*. and Cartón-Garcia *et al*. described the deletion of the *MYO5B* gene in mice and its close phenotypic similarity to the human disease ^39, 40^. The inducible intestine-specific knockdown of *MYO5B* could successfully recapitulate human MVID in just 4 days post induction. However, germline knockdown of *MYO5B* in mice very closely showcases hallmarks of MVID in the duodenum during the gestational stage (day 20 of gestation) and in newborn mice ^40^. In addition, in a recently developed swine model published as an abstract form, where the mutated gene in *MYO5B* ( *P663L*) is introduced, the disease phenotype is similarly discernable ^41^. The pig model is the first large animal model of human MVID that develops diarrhea shortly after birth and may be useful for preclinical studies.

Similar to studies in cell lines and patients with MVID, intestinal tissues from *MYO5B*-knockout mice showed decreased localization of apical protein NHE3 but not CFTR ^42^. Also, the tamoxifen-inducible VilCre ^ERT2^;MYO5B ^flox/flox ^model demonstrated a loss of apical NHE3, sodium glucose transporter-1 (SGLT1), DRA, and aquaporin-7 (AQP7) ^38^. These mice did not show an intestinal barrier defect, based on Ussing chamber analysis, but exhibited decreased SGLT1 activity and increased CFTR activity. However, in MVID patient intestinal explants, increased permeability has been reported ^43^. Also, mislocalization of CFTR was demonstrated in some patient biopsies ^34^. These differences further highlight that knockout of *myo5B* may not necessarily resemble the presence of a mutated MYO5B protein. In addition, it is unclear if these models have defects in the large intestine, as most of the studies have utilized the small intestine alone.

### Enteroids derived from models of microvillus inclusion disease

Intestinal enteroids have recently emerged as an important model which closely recapitulates the human disease phenotype due to epithelial defects. Because of the presence of all types of epithelial cells and the self-renewing capacity of the enteroids, these cultured native intestinal epithelial cells represent a superior model as compared to cancer cell lines. In this regard, there is a significant scarcity of patient-derived enteroids from MVID ^13^. This is mainly due to the lack of a reasonably large patient cohort and the very early onset and fatality of the disease. However, intestinal enteroids generated from different mouse models where *MYO5B* is knocked down exhibited abnormalities with features similar to those seen in the small intestinal tissues of MVID patients ^33, 38, 42^. A recent study conducted by Mosa *et al.* underscored the importance of studying the pathology of MVID by demonstrating the ability to rescue the defects present in MUNC18-2 (mutated in FHL5) knockdown mouse enteroids by expressing the human WT protein and not by the mutant FHL5 patient variant (P477L) ^36, 44^. It is noteworthy to mention that owing to the rare nature of the enteropathy, long-term preservation of patient samples to generate organoids is warranted to enhance the current understanding of the disease.

### Caenorhabditis elegans nematode model

Although very simple, consisting of only a few enterocytes, the *C. elegans* nematode model possesses a close resemblance to human intestinal epithelium with distinct polarization of apical and basolateral membranes with a prominent microvillus brush border. In this regard, by silencing various components in the V-ATPase complex (an important regulator of cellular trafficking), the authors identified that specific subunits of the protein complex, in particular V0, are upstream of other genetic defects which leads to a MVID-like phenotype in this model ^45^. Due to the simplicity of the model, this may be important for use as a platform to study the development of the disease as well as potential cellular mechanisms, which can be a target for developing drug molecules for MVID management.

## Extraintestinal manifestations in microvillus inclusion disease

The *MYO5B* gene is expressed in all epithelial tissues, but the most prominent phenotype is observed in the intestine. However, several extraintestinal pathologies have also been reported in other tissues. In this regard, pathologies identified include renal Fanconi syndrome, cholestasis, hematuria, and pneumonia ^27, 46^. Therefore, animal models of MVID could be useful to study these conditions that may be missed in humans owing to the complications associated with disease diagnosis, the very early onset, and lack of survival. With respect to biliary dysfunction, a recent study found cholestasis in 30% of their patient cohort, which was characterized by a low level of serum gamma-glutamyl transpeptidase (GGT) ^47^. The study reported abnormalities in the recycling of MYO5B and RAB11A and mistargeting of bile salt export pump (BSEP) to the canalicular membrane of hepatocytes. Although cholestasis in MVID patients was previously thought to be solely due to TPN-related toxicity, evidence has emerged supporting cholestasis in the absence of TPN due to apical trafficking defects in MVID hepatocytes ^48^. In this regard, the investigators noted that the unexpected low levels of GGT in MVID patients contrasted with the high levels of this surrogate in cases of liver failure associated with TPN. In a very recent preliminary study conducted in *MYO5B* null mice and pigs with Navajo mutation (published in an abstract form), the authors demonstrated an interference with apical membrane trafficking in hepatocytes. Specifically, multidrug resistance associated protein-2 (MRP2) and BSEP were mislocalized to subapical compartments. In addition, dipeptidyl peptidase-4 (DPPIV) enzyme was mistrafficked and the liver bile canaliculi lacked branching, highlighting the importance of *MYO5B* in studying liver dysfunction associated with MVID patients ^49^.

## Conclusions

Malabsorptive disorders lead to retarded growth and nutritional deficiencies. The complex nature of these disorders poses a challenge for treatment options ^50^. Understanding the pathophysiological mechanisms of malabsorption should improve current management protocols and immensely enhance our knowledge regarding intestinal physiology. In this regard, increased understanding of the intriguing malabsorptive disorders of childhood such as MVID should offer new insights at the cellular and molecular levels to unravel the link between cellular trafficking and epithelial absorptive processes. The research in the field of MVID has considerably progressed over the last decade. The generation of novel mouse models with *MYO5B* deletion has been successful in recapitulating various hallmark features of MVID. So far, the utilization of these models has not only substantiated the role of *MYO5B* and trafficking machinery in the disease’s pathogenesis but also underscored the importance of cellular trafficking mechanisms in maintaining optimal function of nutrient and electrolyte transporters such as SGLT1 and NHE3. Unlike the *in vitro* and *in vivo* mouse models, where loss of *MYO5B* ideally disrupts intracellular trafficking in all cells, the manifestation of abnormalities in MVID patients is patchy and sometimes confined to a few enterocytes ^5^. In addition, although some studies described the presence of abnormalities in the colon and rectum of MVID patients, most animal models focused only on the duodenum and upper small intestine ^35, 38, 39, 42^. More studies in the distal parts of the small intestine and colon should broaden our understanding of the compensatory mechanisms that the intestine may employ to adapt in consequences of *MYO5B* mutations. The mechanisms underlying lipid malabsorption associated with MVID remain elusive. Therefore, investigations to explore the molecular basis for dysregulation of lipid absorption in MVID patients and mouse models are warranted. The inducible *MYO5B-*deficient mouse models have the additional advantage of studying the consequences of time- and age-dependent occurrences of disease-specific hallmarks ^29, 33, 38^. Although MVID is a rare disorder, the organoids derived from MVID patients can provide unique opportunities to model the disease and modify the mutated genes by state-of-the-art approaches, including the CRISPR/Cas9 gene editing system, for rescuing the defective phenotype ^29^.
